# Association Between Insufficient Sleep, Depressive Symptoms, and Suicidality Among Florida High School Students

**DOI:** 10.5888/pcd20.220403

**Published:** 2023-07-13

**Authors:** Justine Gunderson, Katherine McDaniel, Alexis DiBlanda

**Affiliations:** 1 Florida Department of Health, Public Health Research Unit, Division of Community Health Promotion, Tallahassee, Florida; 2 Florida State University, College of Medicine, Tallahassee, Florida

## Abstract

**Introduction:**

Emerging research suggests insufficient sleep can heighten the risk of poor mental health among adolescents. We examined the relationship between sleep, depressive symptoms, and suicidal thoughts and behaviors among Florida high school students.

**Methods:**

Data were from the 2021 Florida High School Youth Risk Behavior Survey, a statewide representative sample of students in grades 9 to 12. We estimated prevalence of 1 indicator of depressive symptoms — feeling sad or hopeless for 2 or more weeks — and 2 indicators of suicidality — having considered attempting suicide and making a suicide plan — by insufficient sleep status (<8 h of sleep on an average school night). Multivariate logistic regressions were used to calculate odds ratios measuring the association between insufficient sleep, poor mental health, and suicidality.

**Results:**

Compared with students reporting sufficient sleep, those with insufficient sleep were more likely to feel sad or hopeless (42.7% vs 28.1%), have considered suicide (19.1% vs 12.5%), or have made a suicide plan (14.8% vs 9.6%). In adjusted multivariate models, compared with students with sufficient sleep, students with insufficient sleep had higher odds of feeling sad or hopeless (adjusted odds ratio [AOR] = 1.83; 95% CI 1.54–2.17), and having made a plan about how they would attempt suicide (AOR = 1.32; 95% CI, 1.00–1.74), but did not have significantly higher odds of having seriously considered suicide.

**Conclusion:**

Increased attention to sleep as a modifiable risk factor for mental health among adolescents is particularly important because of the many less modifiable factors that contribute to mental health difficulties in this population. Adolescent mental health programs should consider sleep an important factor in suicide prevention.

**Summary**

**What is already known on this topic?**

Emerging studies suggest a connection between insufficient sleep and suicidal ideation; however, additional research is needed to further investigate the relationship between sleep and mental health among adolescents.

**What is added by this report?**

This study uses timely, representative, state-level data to examine the relationship between insufficient sleep, suicidal ideation, and depressive symptoms among high school students. After adjustments, we observed disparities in mental health between adolescents with and without insufficient sleep.

**What are the implications for public health practice?**

Findings underscore the importance of addressing the role of sleep as a modifiable risk factor contributing to mental health difficulties among adolescents. Suicide prevention efforts should consider adolescents experiencing chronic sleep deprivation.

## Introduction

Depression and suicidality among adolescents are significant public health concerns. Prevalence estimates from the national 2019 Youth Risk Behavior Survey (YRBS) show that 36.7% of high school students in the US felt sad or hopeless, 18.8% seriously considered attempting suicide, and 15.7% made a suicide plan (1) compared with 33.7%, 15.6%, and 11.8% in Florida, respectively (2). Given that suicide is a leading cause of death among adolescents and young adults (3), it is important to examine potentially modifiable risk factors to decrease future illness and death in this population.

One modifiable risk factor receiving increased attention from researchers is insufficient sleep. Studies have shown that serious health consequences of insufficient sleep among adolescents range from increased risk-taking behaviors, such as substance use and impaired driving, to decreased physical well-being and perceived quality of life (4–6). Other studies found that the consequences of sleep deprivation extend to negative effects on mental health (7,8). One study examined potential links between sleep difficulties and suicidality among high school students and found that adolescents who slept less than 8 hours a night were approximately 3 times more likely to attempt suicide than those who slept 9 or more hours (adjusted odds ratio [AOR] = 2.89; 95% CI, 1.07–7.81) (7). A study using 2017 national YRBS data on adolescents aged 14 years or older found that insufficient sleep, defined as less than 8 hours on an average school night, was associated with increased risk of suicidal ideation (AOR = 1.35; 95% CI, 1.16–1.58) (8).

The relationship between mood disorders and insufficient sleep is known to be bidirectional in adults, and emerging literature suggests a similar pattern may exist among adolescents (9,10): depressive symptoms and suicidal thoughts and behaviors may lead to poor sleep, and poor sleep can increase the risk of depressive symptoms and suicidality. However, recent longitudinal studies among adolescents found that shorter sleep duration at baseline increased the presence of depressive symptoms at follow-up, providing evidence that sleep deprivation precedes mental health difficulties (11,12). This pattern is concerning because the number of US adolescents who get the recommended 8 hours of sleep per night decreased significantly, from 31.1% in 2007 to 22.1% in 2019 (1).

Although studies have documented significant associations between insufficient sleep and suicidal behaviors among adolescents, additional timely research is needed to explore the connections between insufficient sleep and mental health overall, particularly given the recent decline in sufficient sleep among high school students (1) and the increase in depressive symptoms (13,14). The 2021 Florida High School Youth Risk Behavior Survey (FL-YRBS) provided a unique opportunity to use very recent, representative, state-level data to explore the relationship between sleep, depressive symptoms, and suicidal thoughts and behaviors. Such data can help local public health officials better understand these relationships and enable more timely suicide prevention efforts among adolescents.

## Methods

The FL-YRBS is a statewide, school-based, confidential survey of Florida’s public high school students in grades 9 to 12 that collects data on priority health-risk behaviors that contribute substantially to leading causes of death, disability, and social problems among adolescents that may extend into adulthood. The 2021 FL-YRBS was conducted in partnership with the Centers for Disease Control and Prevention (CDC), whose institutional review board approved the protocol for the national YRBS. The FL-YRBS uses a complex, 2-stage cluster probability sample design. First, a random sample of public high schools is selected for participation. Second, within each selected school, a random sample of classrooms is chosen, and all students in the selected classroom sample are eligible to participate. Student participation is voluntary and anonymous. Consent processes vary by school district; however, in 2021, all participating districts obtained parental consent through passive opt-out permission forms. The biennial FL-YRBS was first administered in 2001. In 2021, the most recent year of survey administration, 4,766 students participated, yielding 4,672 completed questionnaires with usable data. The school response rate was 99%, and the student response rate was 64% for an overall response rate of 64%. All responses were weighted to obtain a representative sample of Florida public high school students in grades 9 to 12 (15).

We used 4 survey questions to examine the association between insufficient sleep, depressive symptoms, and suicidality among Florida high school students. Sleep duration was assessed by asking respondents how many hours of sleep they got on an average school night, with response options of 4 hours or less, 5 hours, 6 hours, 7 hours, 8 hours, 9 hours, and 10 or more hours. Following recommendations from CDC and the American Academy of Sleep Medicine, insufficient sleep was defined as reporting less than 8 hours of sleep on an average school night (16,17). Three mental health questions asked respondents whether, in the past 12 months, they 1) felt so sad or hopeless almost every day for 2 weeks in a row or more that they stopped doing usual activities, 2) seriously considered attempting suicide, and 3) made a plan about how they would attempt suicide. The mental health questions were assessed by using dichotomous yes and no response options.

We adjusted for the following demographic characteristics in multivariate models: sex, race or ethnicity, and grade level. Sex was coded dichotomously with male serving as the reference category. Participant race or ethnicity was collapsed into the following categories: non-Hispanic White, non-Hispanic Black, Hispanic or multiple Hispanic (includes Hispanic in addition to one or more races), and non-Hispanic Other. Grade level was coded into 9th, 10th, 11th, and 12th, with 9th grade serving as the reference category. In subsequent multivariate models, we controlled for 4 additional possible risk factors for poor mental health (bullying victimization, electronic bullying victimization, current alcohol use, and current marijuana use) and 1 potential protective factor (physical activity). Bullying victimization and substance use indicators were selected for inclusion as risk factors because studies have found school bullying (18), cyberbullying (19), alcohol use (20), and marijuana use (21) to be associated with increased risk of depression and suicidality among adolescents. Physical activity was selected as a protective factor because existing literature has shown that participating in physical activity is associated with decreased odds of depressive symptoms among adolescents (22). Bullying victimization was defined as an affirmative response to the question, “During the past 12 months, have you ever been bullied on school property?” Electronic bullying victimization was defined as an affirmative response to the question, “During the past 12 months, have you ever been electronically bullied? (Count being bullied through texting, Instagram, Facebook, or other social media.).” Current alcohol and marijuana use were defined as use during the 30 days before the survey. Physical activity engagement was based on response to the question, “During the past 7 days, on how many days were you physically active for a total of at least 60 min per day? (Add up all the time you spent in any kind of physical activity that increased your heart rate and made you breathe hard some of the time.).” Respondents who reported physical activity on 5 or more days were coded as physically active.

We conducted univariate, bivariate, and multivariate analyses to examine the relationship between insufficient sleep and mental health. First, descriptive statistics were calculated for all study variables to examine the distribution of sample characteristics. Then we used Rao-Scott modified χ^2^ tests to analyze bivariate associations between insufficient sleep and the 3 mental health indicators. Lastly, the 3 mental health indicators were regressed separately on insufficient sleep by using 2 nested sets of binary logistic regressions. The first set of models controlled for demographic factors, and the second set added in controls for risk and protective factors for poor mental health. To adjust for the strong association between depression and suicidality we controlled for the effects of feeling sad and hopeless on having seriously considered suicide and making a suicide plan in the second set of models. We reported adjusted odds ratios (aORs) and corresponding 95% CIs. Statistical tests were considered significant if *P* values were <.05. We used SAS version 9.4 (SAS Inc) to perform all analyses. SAS survey procedures were used to account for the complex YRBS sampling design and population weights in all analyses.

## Results

In our sample of Florida public high school students in 2021, 39.3% reported having felt so sad or hopeless almost every day for 2 weeks or more in a row that they stopped doing usual activities, 17.7% seriously considered suicide, and 13.8% made a suicide plan (Table 1). Most students (78.4%) reported insufficient sleep on an average school night. The sample was nearly evenly split between males (50.9%) and females (49.1%) and by grade level, with 26.5% in 9th grade, 25.5% in 10th grade, 24.3% in 11th grade, and 23.6% in 12th grade. Non-Hispanic White students made up the largest group (37.5%), followed by Hispanic or multiple Hispanic students (34.3%), non-Hispanic Black students (21.4%), and non-Hispanic Other students (6.7%). Approximately 1 of every 8 students reported in the past 12 months experiencing bullying on school property (12.7%) and 12.9% reported experiencing electronic bullying. Almost 1 in 4 (23.7%) reported current alcohol use, and 17.2% reported current marijuana use. Nearly two-thirds of students (60.7%) were classified as physically inactive.

**Table 1:** Sample Characteristics of Participants (N = 4,672), 2021 Florida Youth Risk Behavior Survey^a^

Variable	N	Weighted % (95% CI)
**Felt sad or hopeless^b^**		
Yes	1,783	39.3 (36.8–41.8)
No	2,829	60.7 (58.2–63.2)
**Seriously considered suicide^c^**		
Yes	805	17.7 (16.0–19.4)
No	3,798	82.3 (80.6–84.0)
**Made suicide plan^c^**		
Yes	632	13.8 (12.5–15.2)
No	3,966	86.2 (84.8–87.5)
**Insufficient sleep^d^**		
Yes	3,449	78.4 (76.8–80.1)
No	979	21.6 (19.9–23.2)
**Sex**		
Male	2,452	50.9 (49.1–52.8)
Female	2,189	49.1 (47.2–50.9)
**Race or ethnicity**		
Non-Hispanic White	1,749	37.5 (32.4–42.6)
Non-Hispanic Black	786	21.4 (17.3–25.5)
Hispanic or multiple Hispanic^e^	1,675	34.3 (29.7–39.0)
Non-Hispanic Other^f^	410	6.7 (5.6–7.8)
**Grade level**		
9	1,600	26.5 (23.6–29.4)
10	1,208	25.5 (23.4–27.7)
11	1,015	24.3 (22.1–26.6)
12	800	23.6 (20.4–26.8)
**Bullying victimization^g^**		
Yes	595	12.7 (11.1–14.3)
No	4,054	87.3 (85.7–88.6)
**Electronic bullying victimization^h^**		
Yes	603	12.9 (11.4–14.3)
No	4,043	87.1 (85.7–88.6)
**Current alcohol use^i^**		
Yes	1,001	23.7 (21.4–25.9)
No	3,497	76.3 (74.1–78.6)
**Current marijuana use^i^**		
Yes	712	17.2 (15.7–18.7)
No	3,843	82.8 (81.3–84.3)
**Physical activity^j^**		
Yes	1,772	39.3 (37.0–41.5)
No	2,691	60.7 (58.5–63.0)

a The reference source used in the study, https://www.floridahealth.gov/statistics-and-data/survey-data/florida-youth-survey/youth-risk-behavior-survey/index.html,is not currently available. For references 2 and 15 (2,15), an alternative data source from CDC is provided with the same data set as the one used in the study.

b Almost every day for 2 or more weeks in a row during the past 12 months.

c During the past 12 months.

d Defined as less than 8 h on an average school night.

e Multiple Hispanic includes Hispanic in addition to one or more races.

f Includes American Indian or Alaska Native, Asian, Native Hawaiian or Other Pacific Islander, and multiple races.

g Defined as having been bullied on school property during the past 12 months.

h Defined as having been bullied through texting, Instagram, Facebook, or other social media during the past 12 months.

i Use during previous 30 days before survey administration.

j Defined as having been physically active for 60 min or more per day on 5 or more days during the past 7 days.

Significant differences were found when we analyzed all 3 depressive symptoms and suicidality indicators by sleep status (Table 2). Compared with students reporting sufficient sleep (≥8 h), students who reported insufficient sleep (<8 h) were more likely to report feeling so sad or hopeless almost every day for 2 weeks or more in a row that they stopped doing usual activities (42.7% vs 28.1%; *P* < .001). Students with insufficient sleep were more likely to report having seriously considered attempting suicide (19.1% vs 12.5%; *P* < .001) and having made a plan about how they would commit suicide (14.8% vs 9.6%; *P* < .001), compared with those reporting sufficient sleep.

**Table 2:** Depressive Symptoms and Suicidality Outcomes by Sample Characteristics Among Participants (N = 4,672), 2021 Florida Youth Risk Behavior Survey^a^

Characteristic	Felt sad or hopeless^b^		Seriously considered suicide^c^		Made suicide plan^c^	
	N (weighted %) [95% CI]	*P* value	N (weighted %) [95% CI]	*P* value	N (weighted %) [95% CI]	*P* value
**Insufficient sleep^d^**						
Yes	1,442 (42.7) [39.9–45.5]	<.001	648 (19.1) [17.2–20.9]	<.001	504 (14.8) [13.2–16.4]	<.001
No	264 (28.1) [24.2–32.0]		119 (12.5) [10.4–14.6]		90 (9.6) [7.6–11.6]	
**Sex**						
Male	653 (27.3) [25.2–29.4]	<.001	271 (11.3) [9.9–12.7]	<.001	209 (8.4) [7.2–9.6]	<.001
Female	1,116 (51.6) [47.8–55.3]		523 (24.1) [21.0–27.1]		416 (19.3) [17.2–21.5]	
**Race or ethnicity**						
Non-Hispanic White	665 (39.5) [35.8–43.1]	.02	299 (18.0) [15.6–20.4]	.002	209 (12.5) [10.5–14.6]	.02
Non-Hispanic Black	272 (35.0) [31.5–38.6]		117 (14.5) [11.9–17.1]		100 (12.6) [10.0–15.3]	
Hispanic or multiple Hispanic^e^	655 (41.4) [38.4–44.4]		281 (18.1) [15.7–20.5]		237 (15.0) [12.9–17.2]	
Non-Hispanic other^f^	174 (42.8) [36.3–49.3]		100 (24.3) [19.2–29.3]		80 (19.0) [14.4–23.6]	
**Grade level**						
9	597 (37.5) [33.7–41.3]	.16	282 (18.1) [15.7–20.5]	.78	236 (15.9) [13.1–18.6]	.03
10	487 (41.4) [38.1–44.6]		213 (18.1) [15.6–20.7]		174 (15.2) [12.7–17.7]	
11	397 (40.7) [37.6–43.9]		173 (17.7) [14.7–20.6]		118 (12.0) [9.8–14.1]	
12	285 (37.8) [33.7–41.9]		124 (16.5) [13.5–19.5]		91 (11.7) [9.0–14.4]	
**Bullying victimization^g^**						
Yes	395 (67.4) [63.2–71.6]	<.001	233 (38.9) [34.2–43.7]	<.001	197 (33.1) [28.9–37.3]	<.001
No	1,377 (35.1) [32.7–37.5]		569 (14.6) [13.1–16.1]		431 (11.0) [9.7–12.3]	
**Electronic bullying victimization^h^**						
Yes	399 (67.8) [63.0–72.7]	<.001	237 (40.4) [36.2–44.5]	<.001	193 (32.1) [27.9–36.3]	<.001
No	1,376 (35.0) [32.6–37.5]		567 (14.4) [12.9–15.9]		437 (11.2) [9.9–12.4]	
**Current alcohol use^i^**						
Yes	528 (53.3) [49.2–57.4]	<.001	308 (30.7) [26.9–34.6]	<.001	244 (24.0) [20.5–27.5]	<.001
No	1,190 (34.8) [32.4–37.3]		472 (13.7) [12.1–15.2]		359 (10.4) [9.2-11.6]	
**Current marijuana use^i^**						
Yes	420 (58.2) [54.5–61.9]	<.001	239 (32.0) [28.2–35.7]	<.001	200 (26.9) [23.1–30.7]	<.001
No	1,333 (35.5) [33.3–37.8]		544 (14.5) [13.1–15.9]		414 (10.9) [9.8–12.1]	
**Physical activity^j^**						
Yes	563 (32.3) [29.2–35.4]	<.001	234 (13.0) [11.2–14.7]	<.001	172 (9.1) [7.5–10.8]	<.001
No	1,150 (43.9) [40.9–47.0]		539 (20.7) [18.4–22.9]		427 (16.5) [14.5–18.5]	

a The reference source used in the study, https://www.floridahealth.gov/statistics-and-data/survey-data/florida-youth-survey/youth-risk-behavior-survey/index.html,is not currently available. For references 2 and 15 (2,15), an alternative data source from CDC is provided with the same data set as the one used in the study.

b Almost every day for 2 or more weeks in a row during the past 12 months.

c During the past 12 months.

d Defined as less than 8 hours on an average school night.

e Multiple Hispanic includes Hispanic in addition to one or more races.

f Includes American Indian or Alaska Native, Asian, Native Hawaiian or Other Pacific Islander, and multiple races.

g Defined as having been bullied on school property during the past 12 months.

h Defined has having been bullied through texting, Instagram, Facebook, or other social media during the past 12 months.

i Use during previous 30 days before survey administration.

j Defined as having been physically active for 60 minutes or more per day on 5 or more days during the past 7 days.

We used 2 sets of models to perform multivariate analyses of the association between insufficient sleep, depressive symptoms, and suicidality (Table 3). After controlling for demographic characteristics, Model 1 showed that students who reported insufficient sleep had higher odds of feeling so sad or hopeless almost every day for 2 weeks or more in a row that they stopped doing usual activities (AOR = 1.82; 95% CI, 1.50–2.21), having seriously considered attempting suicide (aOR = 1.59; 95% CI, 1.30–1.95), and having made a suicide attempt plan (AOR = 1.61; 95% CI, 1.29–2.03) compared with students who reported sufficient sleep. After introducing risk and protective factors in Model 2, associations between insufficient sleep and 2 of the 3 outcomes — feeling sad or hopeless and making a suicide plan — remained significant. Compared with students reporting sufficient sleep on an average school night, students who reported insufficient sleep had 1.83 times higher odds (95% CI, 1.54–2.17) of feeling sad or hopeless almost every day for 2 weeks or more in a row and 1.32 times higher odds (95% CI, 1.00–1.74) of having made a suicide plan. However, students who reported insufficient sleep did not have significantly higher odds of having seriously considered suicide compared with their peers who reported sufficient sleep.

**Table 3:** Characteristics of Two Models, Depressive Symptoms and Suicidality Outcomes by Sample Characteristics Among Participants (N = 4,672), 2021 Florida Youth Risk Behavior Survey^a^

Characteristic	Felt sad or hopeless^b^	Seriously considered suicide^c^	Made suicide plan^c^
	AOR (95% CI)	AOR (95% CI)	AOR (95% CI)
**Model 1: Demographic controls**			
**Insufficient sleep^d^**			
Yes	1.82 (1.50–2.21)	1.59 (1.30–1.95)	1.61 (1.29–2.03)
No	1 [Reference]	1 [Reference]	1 [Reference]
**Sex**			
Female	2.82 (2.40–3.31)	2.49 (1.96–3.16)	2.80 (2.32–3.38)
Male	1 [Reference]	1 [Reference]	1 [Reference]
**Race or ethnicity**			
Non-Hispanic Black	0.77 (0.61–0.97)	0.70 (0.55–0.91)	0.89 (0.66–1.21)
Hispanic/Multiple Hispanic	1.04 (0.88–1.23)	0.95 (0.74–1.21)	1.16 (0.89–1.50)
Non-Hispanic Other^e^	1.07 (0.83–1.36)	1.35 (1.00–1.82)	1.55 (1.14–2.11)
Non-Hispanic White	1 [Reference]	1 [Reference]	1 [Reference]
**Grade level**			
10	1.23 (0.99–1.52)	0.99 (0.77–1.28)	0.98 (0.68–1.41)
11	1.13 (0.97–1.33)	0.93 (0.73–1.20)	0.72 (0.52–0.98)
12	0.98 (0.81–1.19)	0.84 (0.63–1.12)	0.67 (0.46–0.98)
9	1 [Reference]	1 [Reference]	1 [Reference]
**Model 2: Demographic controls and risk/protective factors**			
**Insufficient sleep^d^**			
Yes	1.83 (1.54–2.17)	1.19 (0.90–1.57)	1.32 (1.00–1.74)
No	1 [Reference]	1 [Reference]	1 [Reference]
**Sex**			
Female	2.52 (2.14–2.95)	1.37 (1.07–1.76)	1.62 (1.29–2.04)
Male	1 [Reference]	1 [Reference]	1 [Reference]
**Race or ethnicity**			
Non-Hispanic Black	0.88 (0.71–1.09)	0.85 (0.64–1.12)	1.08 (0.80–1.46)
Hispanic/Multiple Hispanic^e^	1.14 (0.95–1.37)	1.00 (0.74–1.34)	1.20 (0.89–1.63)
Non-Hispanic other^f^	1.08 (0.85–1.37)	1.40 (0.93–2.09)	1.50 (1.03–2.19)
Non-Hispanic White	1 [Reference]	1 [Reference]	1 [Reference]
**Grade level**			
10	1.23 (0.99–1.54)	0.85 (0.64–1.12–	0.88 (0.59–1.30)
11	1.15 (0.99–1.34)	0.83 (0.62–1.11)	0.61 (0.42–0.87)
12	0.92 (0.75–1.13)	0.77 (0.53–1.12)	0.60 (0.39–0.92)
9	1 [Reference]	1 [Reference]	1 [Reference]
**Bullying victimization^g^**			
Yes	2.61 (2.03–3.37)	1.56 (1.16–2.09)	1.91 (1.42–2.56)
No	1 [Reference]	1 [Reference]	1 [Reference]
**Electronic bullying victimization^h^**			
Yes	2.11 (1.57–2.84)	1.69 (1.30–2.20)	1.33 (0.98–1.81)
No	1 [Reference]	1 [Reference]	1 [Reference]
**Current alcohol use^i^**			
Yes	1.54 (1.27–1.86)	1.68 (1.26–2.24)	1.62 (1.19–2.21)
No	1 [Reference]	1 [Reference]	1 [Reference]
**Current marijuana use^i^**			
Yes	1.95 (1.66–2.29)	1.44 (1.12–1.85)	1.60 (1.14–2.23)
No	1 [Reference]	1 [Reference]	1 [Reference]
**Felt sad or hopeless^b^**			
Yes	—j	13.38 (9.51–18.83)	8.50 (5.48–13.18)
No	—j	1 [Reference]	1 [Reference]
**Physical activity^k^**			
Yes	0.71 (0.59–0.86)	0.77 (0.61–0.98)	0.70 (0.53–0.93)
No	1 [Reference]	1 [Reference]	1 [Reference]

Abbreviation: AOR, adjusted odds ratio.

a The reference source used in the study, https://www.floridahealth.gov/statistics-and-data/survey-data/florida-youth-survey/youth-risk-behavior-survey/index.html,is not currently available. For references 2 and 15 (2,15), an alternative data source from CDC is provided with the same data set as the one used in the study. Values are AOR (95% CI).

b Almost every day for 2 weeks or more in a row during the past 12 months.

c During the past 12 months.

d Defined as less than 8 hours on an average school night.

e Multiple Hispanic includes Hispanic in addition to one or more races.

f Includes American Indian or Alaska Native, Asian, Native Hawaiian or Other Pacific Islander, and multiple races.

g Defined as having been bullied on school property during the past 12 months.

h Defined has having been bullied through texting, Instagram, Facebook, or other social media during the past 12 months.

i Use in 30 days before survey administration.

j Included as dependent variable.

k Defined as having been physically active for 60 minutes or more per day on 5 or more days during the past 7 days.

## Discussion

Our findings extend previous research on the relationship between sleep and mental health among adolescents by exploring the association between insufficient sleep and 3 indicators of depression and suicidality — feeling sad or hopeless, having seriously considered suicide, and having made a suicide attempt plan — among a current cohort of Florida high school students. We found that over three-quarters (78.4%) of students did not achieve 8 or more hours of sleep during an average school night. Furthermore, more than 1 in 3 students (39.3%) felt so sad or hopeless they stopped doing usual activities, 17.7% seriously considered suicide, and 13.8% made a suicide plan. Our models demonstrated that insufficient sleep remained a significant predictor of depressive symptoms and suicide planning after controlling for student characteristics and risk and protective factors. These findings are particularly concerning given the large number of students in our sample reporting insufficient sleep.

Our study builds on research reported in a recent study by using national YRBS data documenting significant associations between suicidal thoughts and insufficient sleep (8) by including measures of depressive symptoms and suicide planning. Yet, unlike that study, insufficient sleep was not significantly associated with seriously considering suicide in our sample after adjusting for risk and protective factors. This finding aligns with past studies demonstrating that depressive symptoms explained the relationship between insufficient sleep and suicidal ideation (4,23). Given these inconsistent findings, additional research capturing a broader range of outcomes, including anxiety and resilience, is needed to fully investigate the implications of insufficient sleep on mental health. Increased attention to the effect of insufficient sleep as an adjustable risk factor is particularly important because of the many less-modifiable factors that contribute to mental health difficulties. High school students are at particular risk of habitual sleep loss resulting from unhealthy sleep behaviors, such as excessive caffeine intake, electronic media use, and environmental factors, particularly early school start times (9). Adjusting school start times may help address the relationship between insufficient sleep and poor mental health among young people. Research has demonstrated that students attending schools with early start times have higher odds of attempting suicide (24), whereas delaying school start times significantly increased sleep length on school nights (9,24). Public health practitioners should consider the importance of modifying school start times when reviewing the effects of insufficient sleep on mental health.

Our study had several limitations. First, our data are cross-sectional, which limits the ability to draw causal inferences between insufficient sleep, depressive symptoms, and suicidality. Given the bidirectional nature of the relationship between insufficient sleep and poor mental health, depression, or suicidal ideation may exacerbate sleep problems among adolescents, with insufficient sleep manifesting as a symptom or a separate co-occurring condition. Thus, some students may experience poor mental health before the onset of sleep problems. However, recent longitudinal studies exploring the temporal nature of the relationship have demonstrated that sleep loss can increase risk of depression among adolescents, documenting that shorter sleep durations increased depressive symptoms at follow-up (11,24). Future studies using longitudinal and prospective methods are warranted to fully examine the multiple pathways from insufficient sleep to depressive symptoms and suicidality among adolescents. Second, FL-YRBS data are self-reported, which has the potential of response and recall bias. Students may respond inaccurately to questions — especially sensitive questions regarding suicidal ideation and mental health — for various reasons, including a desire to provide socially desirable answers, fear of disclosing mental health concerns, or an inaccurate recollection of previous emotional states or events. This could lead to underreporting of depressive symptoms and suicidality; however, all data were collected anonymously to reduce bias. Third, although we adjusted for several important factors known to influence depressive symptoms and suicidality, we were unable to adjust for other possible confounders, such as family structure and socioeconomic status, because FL-YRBS does not collect these data. Lastly, findings can only be generalized to the overall population of Florida students attending public high schools.

In summary, our study demonstrates that Florida high school students who regularly experience inadequate sleep on an average school night are significantly more likely to experience poor mental health, including increased odds of feelings of sadness and hopelessness and suicide planning — a pattern that persisted even after controlling for demographic and other known risk and protective factors. These findings can inform population-based approaches to promote sleep health among adolescents and may represent an important aim in suicide prevention initiatives.
